# Binaural detection thresholds and audio quality of speech and music signals in complex acoustic environments

**DOI:** 10.3389/fpsyg.2022.994047

**Published:** 2022-11-24

**Authors:** Thomas Biberger, Stephan D. Ewert

**Affiliations:** Department of Medical Physics and Acoustics and Cluster of Excellence Hearing4all, University of Oldenburg, Oldenburg, Germany

**Keywords:** audio quality, detection thresholds, complex acoustic environments, auditory modeling, reverberation

## Abstract

Every-day acoustical environments are often complex, typically comprising one attended target sound in the presence of interfering sounds (e.g., disturbing conversations) and reverberation. Here we assessed binaural detection thresholds and (supra-threshold) binaural audio quality ratings of four distortions types: spectral ripples, non-linear saturation, intensity and spatial modifications applied to speech, guitar, and noise targets in such complex acoustic environments (CAEs). The target and (up to) two masker sounds were either co-located as if contained in a common audio stream, or were spatially separated as if originating from different sound sources. The amount of reverberation was systematically varied. Masker and reverberation had a significant effect on the distortion-detection thresholds of speech signals. Quality ratings were affected by reverberation, whereas the effect of maskers depended on the distortion. The results suggest that detection thresholds and quality ratings for distorted speech in anechoic conditions are also valid for rooms with mild reverberation, but not for moderate reverberation. Furthermore, for spectral ripples, a significant relationship between the listeners’ individual detection thresholds and quality ratings was found. The current results provide baseline data for detection thresholds and audio quality ratings of different distortions of a target sound in CAEs, supporting the future development of binaural auditory models.

## Introduction

In daily life, a sound attended to (target) is often interfered with other (masking) sounds as well as by sound reflections and reverberation in enclosed spaces (referred to as a complex acoustic environment, CAE). However, in psychoacoustics, masking is typically assessed under optimal (anechoic) conditions, using abstracted and simplified stimuli (see, e.g., Ewert, 2020), such as pure tones and stationary noise. Such stimuli are suited for investigating basic sensory abilities and limitations of the auditory system, while minimizing cognitive aspects. Here, additional energetic masking (EM), caused by spectral and temporal overlap of the target and the masker in the auditory periphery, plays an important role, degrading the internal representation of the target. In reverberation, additional self-masking and overlap-masking elicited by early and late room reflections (e.g., Bolt and MacDonald, 1949) occur.

Psychoacoustic (e.g., Kopčo and Shinn-Cunningham, 2003) and speech intelligibility (e.g., Cherry, 1953; Best et al., 2015; Ewert et al., 2017) studies showed that in comparison to a co-located condition, listeners benefit from spatially separated target and maskers, referred to as spatial release from masking (SRM). SRM was reduced in echoic environments (e.g., Plomp, 1976; Lavandier and Culling, 2010; Biberger and Ewert, 2019). Reverberation degrades binaural cues (e.g., Rakerd and Hartmann, 1985; Rakerd and Hartmann, 1986) such as interaural level and time differences (ILDs and ITDs). Moreover, amplitude modulations are reduced in the presence of reverberation, lowering the chance listening into the dips of fluctuating masker signals (Houtgast et al., 1980), where EM of the target is lowest.

In many situations, target sounds are transmitted by electroacoustic systems, e.g., a TV set, conference system or earphones, typically involving audio-signal processing. In this case, linear and non-linear distortions introduced by the signal processing and the transmission chain might be perceptible, affecting the perceived audio quality. Accordingly, detectability, as well as the supra-threshold salience of such distortions, are of interest. Comparable to fundamental psychoacoustic research, the consequences of different distortions on audio quality have often been examined under optimal conditions, without maskers and reverberation, including for the development and evaluation of instrumental quality measures (e.g., van Buuren et al., 1999; Moore and Tan, 2003; Tan et al., 2003; Fleßner et al., 2017, 2019). Only a few studies (e.g., Toole and Olive, 1988; Schobben and van de Par, 2004; Schepker et al., 2019) examined the influence of reverberation on the detectability of signal distortions. Toole and Olive (1988) observed a better detectability of signal resonances in reverberant rooms compared to anechoic conditions. Schobben and van de Par (2004) examined the effect of reverberation and loudspeaker cross-talk on the subjective quality of low-bitrate audio coding. They found reduced audibility of coding artifacts in reverberation. Schepker et al. (2019) evaluated the audio quality of a hearing device prototype, aiming at acoustical transparency (i.e., without any perceptible distortion) in rooms with different reverberation times. No large effect of reverberation time was found, suggesting that the use of only a single or few reverberation times might be sufficient for the audio quality assessment of such devices. Only a few approaches, e.g., Cauchi et al. (2019), and Biberger et al. (2021) considered aspects of reverberation affecting quality predictions. Biberger et al. (2021) found monaural spectral cues, capturing spectral coloration distortions of hearing devices aiming at acoustically transparency, to be more reliable for quality predictions in reverberation than cues based on the temporal fine structure or cepstrum correlation.

One other important aspect in CAEs is the number of spatially distributed sound sources (e.g., Weisser et al., 2019; Fichna et al., 2021) interfering with a target sound. However, neither the effect of interfering sounds on the perceived audio quality of a target sound, nor the applicability of existing instrumental audio quality measures to CAEs have yet been systematically examined. Instrumental quality measures have mainly been applied under anechoic conditions without maskers (Beerends et al., 2002; Moore and Tan, 2004; Huber and Kollmeier, 2006; Kates and Arehart, 2010; Harlander et al., 2014). Some auditory perception models have been applied to isolated aspects of CAEs. One example is the (monaural) Generalized Power Spectrum Model (GPSM), which has been applied to psychoacoustic masking with simplified psychoacoustic stimuli (Biberger and Ewert, 2016, 2017) as well as to audio quality for various distortions in anechoic and echoic conditions without maskers (Biberger et al., 2018, 2021).

Overall, relatively little is known about the detectability of distortions and (supra-threshold) audio quality perception in CAEs. It is unclear whether the results of “classical” quality measurements in anechoic conditions can be transferred to acoustic environments of different complexity, and whether existing audio quality models can be straightforwardly applied.

This study investigates the detectability and supra-threshold perception of a variety of prototypical audio signal distortions in CAEs of different complexity: The effect of room reverberation was assessed by using an anechoic (reference) and two echoic rooms with mild and moderate reverberation times (T60) of 0.35 s (resembling a typical living room) and 1.5 s (resembling a larger auditorium, parking lot, or church). The effect of maskers was assessed by configurations with no (reference), one, and two maskers that were either spatially co-located with the frontal target, or spatially separated to both sides of the target. Four types of distortions were applied to the target signal: i) spectral ripples (linear distortion), ii) a saturating, instantaneous non-linearity (non-linear distortion), iii) differences in the target sound-source intensity, and iv) a variation of the spatial position of the target (azimuthal direction of 0°, 4°, and 30° relative to the listener’s viewing direction). The target was either speech, an acoustic guitar (representing a musical instrument), and a pink noise (representing environmental background noise). These targets differ in their spectro-temporal characteristics and might be differently affected by the distortions. While the acoustic guitar shows strong transients, the pink noise is stationary and produces a broadband excitation of auditory filters more equally than speech and the guitar. Speech was considered as the most relevant target in daily life and thus applied to all experiments of this study, while guitar music and noise were only applied to a subset of experiments. The International Speech Test Signal (ISTS; Holube et al., 2010) and a pop music excerpt were used as maskers, reflecting typical (disturbing) sounds in CAEs.

In the first experiment, detection thresholds for distorted signals were measured for a subset of the conditions, while in the second experiment, supra-threshold audio quality ratings were obtained for two different degrees of distortion. Based on the systematic data set obtained, it was investigated (a) whether room reverberation and masker configuration affects detection thresholds and quality ratings for distorted signals; (b) whether distortion-detection thresholds and quality ratings are related, allowing adjustments of signal processing, as well as individualized perception models, based only on distortion-detection thresholds; (c) whether the individual listeners’ overall performance to detect or rate the target distortions is correlated across conditions having different amount of reverberation and maskers; (d) the extent to which existing auditory models are applicable to distortion detection and audio quality ratings in such CAEs.

## Materials and methods

### Listeners

Sixteen self-reported normal-hearing listeners (7 female, 9 male) with a mean age of 28.7 years (all native German speakers) participated in the experiments. Ten of the sixteen participants received an hourly compensation. The other participants were employed by the Department of Medical Physics and Acoustics at the University of Oldenburg. All listeners had prior experience in psychoacoustic measurements.

### Stimuli

#### Target and masker signals

German speech (spoken language), acoustic guitar, and pink-noise stimuli from the study of Fleßner et al. (2019), having different spectro-temporal properties, were used as target. The speech stimulus “ein Haus, keine Brücke” (“a house, no bridge”) was spoken by a female speaker. The speech stimulus shows slow amplitude modulations (5-Hz range) and a relatively narrowband spectrum. The excerpt of a guitar piece comprised many transients and a wider bandwidth. The pink noise was a stationary stimulus with a broadband spectrum, covering the entire audible frequency range. All target signals had a duration of 2 s.

A male-transformed version of the ISTS speech signal (Holube et al., 2010) as applied in Schubotz et al. (2016) and Ewert et al. (2017) and a pop-music excerpt taken from Fleßner et al. (2019) were used as maskers. ISTS is nonsense speech generated from six different speakers in different languages (American-English, Arabic, Mandarin, French, German, and Spanish). The music signal includes multiple instruments and vocals, with a rather broadband spectrum. The maskers had a duration of 2.5 s and started 0.5 before the target onset. Raised-cosine ramps of 10 ms were applied to the masker and target stimuli. All signals were convolved with binaural room impulse responses (BRIRs) to define their spatial position and to simulate room reverberation (see Section “Rooms and masker configurations”).

#### Target stimulus distortions

The target stimuli were subjected to four different types of distortions; spectral ripples, non-linear saturation, intensity-based, and spatial:

Spectral ripples (linear distortions) were introduced as described in Fleßner et al. (2019), using sinusoidal modulation of the spectral envelope. Ten periods of the spectral sinusoidal modulation were applied between 50 Hz and 16 kHz, with equidistant spacing on a logarithmic frequency axis, corresponding to about 1.2 spectral ripples per octave. The spectral modulation depth (peak-to-valley ratio in dB) was adjusted to change the amount of distortion.

Non-linear distortions caused by a simple instantaneous symmetric saturating input–output (I/O) characteristic (referred to as non-linear saturation) simulated signal distortions caused by, e.g., large displacements of the loudspeaker diaphragm at high signal levels. The I/O characteristic was implemented as $y\left(t\right)=x\left(t\right)-\alpha \times {\left(x\left(t\right)\right)}^{3}$ , where x(t) and y(t) are input and output signals, respectively. The factor α weights the cubic term relative to x(t), and thus controls the nonlinearity of the I/O characteristic. Input values were limited to the range $\pm \sqrt{\frac{1}{3\cdot \alpha }}$ where the non-linear I/O characteristic completely saturates (soft clipping). This saturating I/O function resulted in pronounced harmonic distortions at higher signal levels, typically occurring at signal onsets and transients. These additionally introduced frequency components likely provided spectral or amplitude modulation cues to the listeners.

Intensity-based distortions were introduced by adjusting the overall sound level in dB relative to the level of the reference signal. In contrast to spectral ripples and non-linear saturation, no spectral amplitude modulation cues were introduced.

Spatial (binaural) distortions were introduced by changing the azimuth location of the target using the appropriate BRIRs. The reference target was always presented in front (0° azimuth) of the listeners, while the spatially distorted target was shifted to the right side (relative to the viewing direction of the listener).

Anchor signals were generated by applying a 3.5 kHz low-pass filter, non-linear saturation and spatial distortion to the reference signals. The non-linear saturation (α_speech_ = 0.25, α_music_ = 0.34, α_noise_ = 0.4) and spatial distortion (position at 40° azimuth) in the anchor were more pronounced than the distortions applied in the other stimuli of this study.

For the detection experiment, the strength of distortion was adjusted during the experiment according to the listener’s response (see Section “Apparatus, procedure, and statistical analysis”), while for the quality rating experiments distortions were applied in two different “effect strengths,” denoted as mild and moderate distortions, using the parameters provided in Table 1. For non-linear saturation, Table 1 provides values for the dimensionless parameter α and the maximum total harmonic distortion (THD) for the peak value of the reference signals in percent.

**Table 1:** Experimental parameters (and units) controlling the amount of distortions (columns) in the detection and discrimination experiments and for the quality rating experiments (mild/moderate).

	Spectral ripples (peak-to-valley ratio in dB)	Non-linear saturation (dimensionless parameter α)	Intensity (ΔdB re reference)	Spatial (Δ azimuth ° re reference)
**Detection and discrimination experiments**				
Starting value	18	0.62(33.8)	6.5	18
Initial step size	5	0.2	2	4
Minimum step size	1.5	0.035	0.2	0.3
**Supra-threshold quality ratings**				
Speech	12/18	0.11(15.3)/0.17(21.4)	1.5/4	4°/30°
Guitar	2.5/5	0.18(22.1)/0.28(27.1)	1/4	4°/30°
Noise	8/14	0.18(22.1)/0.37(29.7)	1.5/4.5	4°/30°

For a better representation of the amount of non-linear distortion (second column), the THD@peak-values given in percent are provided in parentheses in addition to the dimensionless parameter α.

#### Rooms and masker configurations

Three room conditions were realized using headphone auralization and BRIRs generated by the room acoustics simulator (RAZR; Wendt et al., 2014). RAZR calculates early reflections up to the third order using the image source model (Allen and Berkley, 1979), while later reflections were calculated by a feedback delay network (Jot and Chaigne, 1991). An assessment of various common room acoustical parameters and subjective ratings of perceived room acoustical attributes showed a good correspondence between simulated and real rooms (see Wendt et al., 2014; Brinkmann et al., 2019).

An anechoic room served as the reference, only providing the direct sound. A small room with dimensions of 5.28 × 3.5 × 2.5 m^3^ (length x width x height) and a room volume of 46 m^3^, was realized with an average reverberation time of T60 of 0.35 s (0.4, 0.37, 0.35, 0.32, and 0.29 s were observed for 0.25, 0.5, 1, 2, and 4 kHz). These parameters were motivated by the average values of reverberation time measurements in furnished living rooms (Díaz and Pedrero, 2005). A large room with dimensions of 7.5 × 4.52 × 3 m^3^ (~100 m^3^) was used with an average T60 of 1.5 s (1.53, 1.53, 1.56, 1.44, and 1.45 s at 0.25, 0.5, 1, 2, and 4 kHz). The volume of the large room is similar to the largest furnished living rooms measured by Díaz and Pedrero (2005) which had on average a room volume of about 95 m^3^ and a T60 of about 0.6 s. The longer T60 of 1.5 s was chosen to better represent environments with pronounced reverberation.

**Figure 1:**
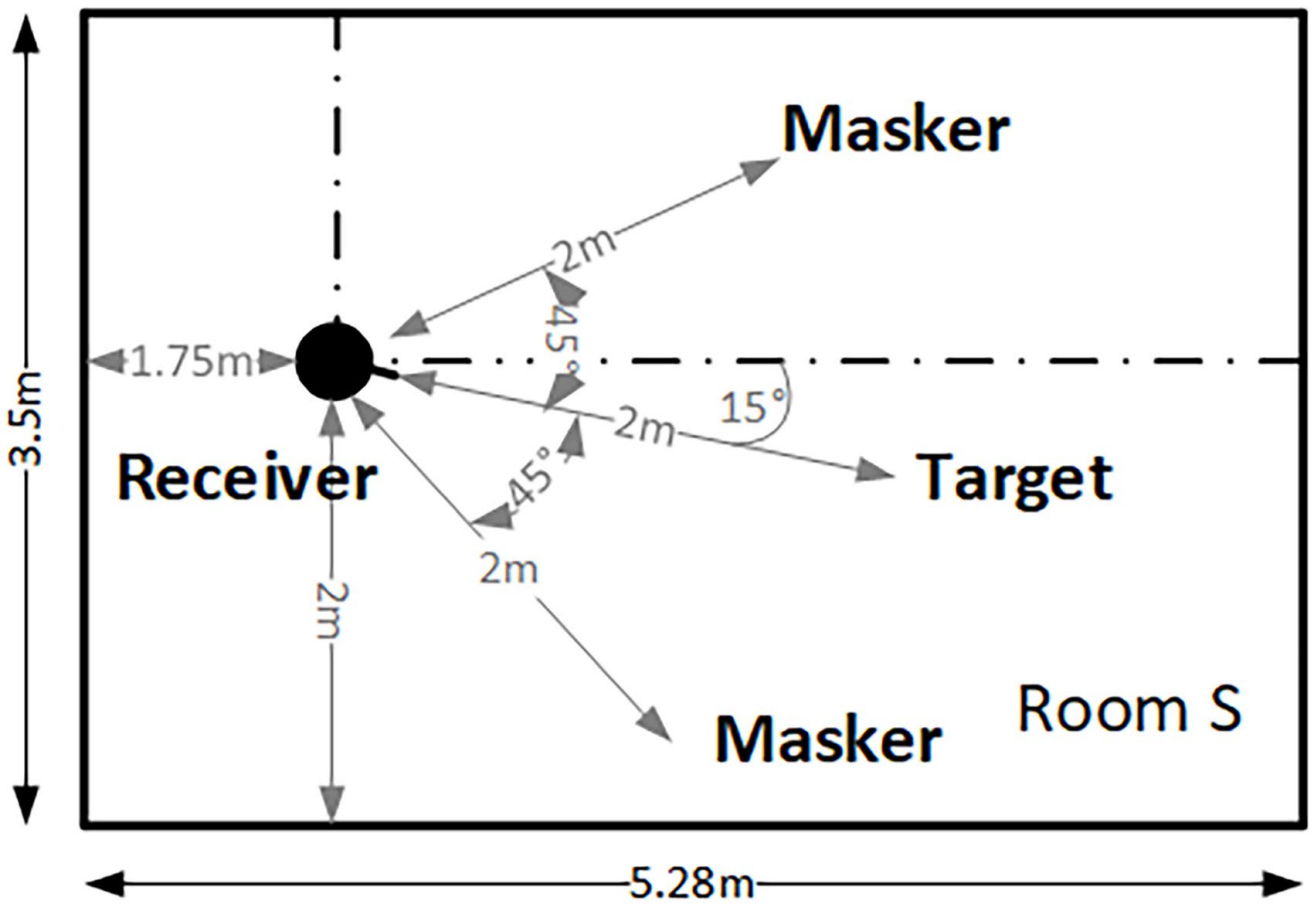
Illustration of the target, receiver, and masker positions in the small room. The same target-receiver-masker positions relative to the lower-left corner as the room origin were used in the anechoic and large room. Accordingly, the distance between the left masker and the upper wall increased from 0.5 m to 1.5 m.

The target and masker sources were convolved with the BRIRs such that they were placed on each of the positions as indicated in Figure 1 for the target and maskers. In each of the three rooms, the receiver and target had identical positions. Different masker configurations were only examined in the small room. Figure 1 illustrates the condition 2M_sep_, with two spatially separated maskers at ±45° azimuth from the target position in the small room. In the 1M_sep_ condition, only the left spatially separated masker was presented. In the co-located masker configuration, 2M_co_, the two maskers were spatially co-located with the target (that always remained in the same position). In the separated conditions, the masker to the left was always the ISTS speech signal, while the masker to the right was always the pop music excerpt. The direct-to-reverberant ratios between target and receiver (DRR_T_), between left masker and receiver (DRR_ML_), and between right masker and receiver (DRR_MR_) are given in Table 2 for all three rooms.

**Table 2:** Room acoustical properties of the three different rooms.

Room	Volume (m^3^)	T60 (s)	DRR_T,0°_ (dB)	DRR_T,4°_ (dB)	DRR_T,30°_ in (dB)	DRR_ML_ in (dB)	DRR_MR_ in (dB)
Anechoic	–	0	∞	∞	∞	∞	∞
Small	46	0.35	−4.7/−4.3	−5.8/−3.5	−11/−1.8	−2.5/−12.8	−13.9/−1.6
Large	100	1.5	−6.7/−6.3	−7.8/−5.5	−13.5/−3.7	−3.1/−15.6	−16.6/−2.8

In each room the receiver-target/masker-source distance was 2 m. The third column is the reverberation time T60 in s. All DRRs are provided for the left/right ear. DRR_T,0°_ refers to the target and the co-located maskers placed at 0° azimuth, while DRR_T,4°_ and DRR_T,30°_ refer to the target source positions at 4° and 30° azimuth (spatial distortions). DRR_ML_ and DRR_MR_ refer to the spatially separated masker on the left (ISTS) and right (pop music), respectively. The values of DRR_T,0°_, DRR_T,4°_, and DRR_T,30°_ were calculated from the receiver-target-source BRIRs. DRR_ML_ and DRR_MR_ were calculated from the receiver-left-masker and receiver-right-masker BRIRs.

The receiver-target-masker positions were asymmetrically arranged in the room, with a distance of 2 m between the target/maskers and the receiver. All sources and the receiver were positioned at a height of 1.7 m above the floor. Such an asymmetric arrangement in the room is more likely to occur in daily life than an unnatural, completely symmetrical arrangement. The asymmetric arrangement in the room results in small long-term level differences between the ears caused by early reflections, while no such differences are present for the direct sound. The fixed distance of 2 m, independent of the room, was chosen to represent a typical distance between the receiver and the sound-emitting device, e.g., a TV.

### Apparatus, procedure, and statistical analysis

Listeners performed the experiments with dichotically presented stimuli *via* Sennheiser HD 650 headphones, while seated in a double-walled, sound-attenuated booth. The transfer function of the headphones was digitally equalized to obtain a flat frequency response in the artificial ear (B&K Type 4153). The level of the reference and masker signals at 0° in the anechoic condition was 61 dB sound pressure level (SPL). Depending on the reverberation time of the simulated room and the number of maskers, the overall level could reach up to about 78 dB SPL. Subjects responded *via* a touchscreen. All audio files had a sampling rate of 44.1 kHz.

All listeners started with the detection experiment, where only speech signals were used. A three-alternative, forced-choice (3-AFC) procedure was used to determine distortion-detection thresholds. Three intervals were presented, and listeners had to identify the randomly chosen interval containing the distorted speech signal (target). The strength of the distortion was varied according to a 1-up, 2-down procedure for estimating the 70.7% correct point of the psychometric function (Levitt, 1971). To reduce the measurement time, the 2-s speech target was separated into two 1-s-segments that were randomly selected per trial. Stimuli in each trial were separated by 300-ms silent intervals. The initial and minimum step sizes used in the experiments are provided in Table 1. After the minimum step size was reached, six reversals were measured, from which the mean threshold was calculated. The final threshold was the mean of the estimates from two measurement runs. All measurements were performed using the AFC-framework (Ewert, 2013). The detection experiment was divided into two 45-min sessions. The order of presentation of distortions was Latin-Square balanced, while the order of the room conditions Anechoic, Small, Small,2M_sep_, and Large, was randomized. Prior to the actual measurement, a randomly selected room condition was used as training run for each type of distortion.

For the (supra-threshold) audio-quality ratings, distorted speech, guitar music, and noise were used as the target. A measurement procedure applied in previous studies of Fleßner et al. (2017, 2019) was used, similar to the Multiple Stimulus Test with Hidden Reference and Anchor (MUSHRA, ITU-R, 2014). Listeners had to rate quality differences between several distorted targets, also denoted as test signals, and a given (unprocessed) reference target, by using a numerical rating scale ranging from 0 (“very strong difference”) to 100 (“no difference”). To ensure that listeners used the full range of the rating scale and to test the reliability of the listeners’ ratings, a hidden reference (without any distortions) and a strongly distorted anchor signal were included. The audio signals were played in a loop and the listeners could listen as long as they wished. Listeners could also switch between the different test signals at any time, in which case the audio restarted at the beginning. The quality rating experiment was divided into three sessions: In the first (test) and third (retest) session the *Effect of room* was assessed, and in the second session the *Effect of masker configuration* (test–retest) was assessed. In the *Effect of room* sessions, participants rated audio quality for distorted speech, guitar, and noise targets randomly presented in the Anechoic, Small and the Large room. In the *Effect of masker configuration* session, participants rated distorted speech targets for different configurations of interfering maskers in the Small room. Prior to the actual measurement phase in the first and second session a training run to familiarize the participants with the procedure was performed.

The results of the initial detection experiment were used as the criterion for participation in this study. The mean values of the listener’s detection and discrimination thresholds had to be below the values given in Table 1 for the speech target with mild distortions. Five listeners had intensity JNDs slightly above the intended limit of 1.5 dB, but were included given that they clearly fulfilled the entrance criterion for the other three distortions. In total, nine of 25 initially screened listeners did not pass the criterion, resulting in the 16 participants of this study.

For statistical analysis, repeated-measures analysis of variance (ANOVA) was applied using IBM SPSS. Greenhouse–Geisser correction was applied if sphericity was violated. Bonferroni correction was applied in the post-hoc pairwise comparisons. The effect size of contrasts was calculated as $ES=\sqrt{\left[F\left(1,df\right)\right]/\left[F\left(1,df\right)+df\right]}$ , where *F* and *df* refer to the F-ratio and the residual degrees of freedom, respectively.

## Results

### Detection and discrimination thresholds

In the following, the mean distortion detection thresholds and discrimination JNDs for the speech target based on the average across sixteen listeners are reported.

**Figure 2:**
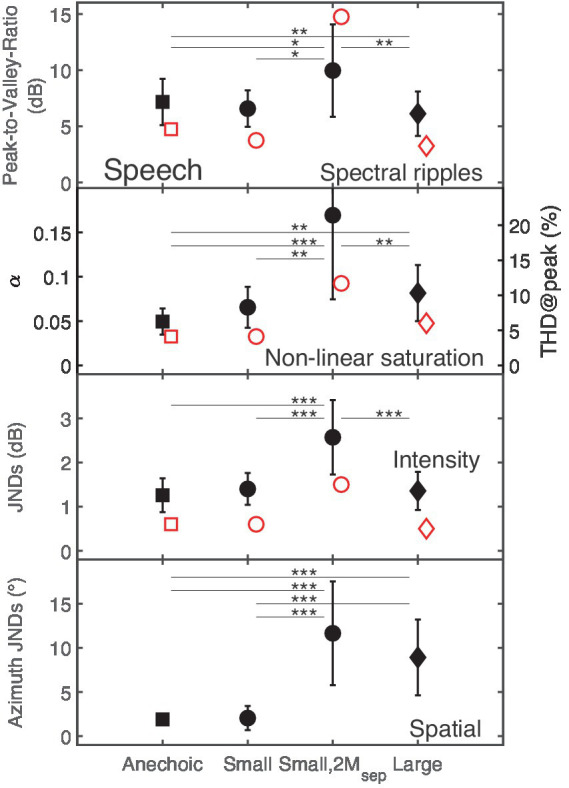
The panels show detection thresholds (black closed symbols) for the four types of distortions in the speech target presented without reverberation (Anechoic room), with mild reverberation (Small room), with mild reverberation and two maskers (Small,2M_sep_), and with moderate reverberation (Large room). The rooms are represented by squares, circles, and diamonds, respectively. Here and in the following figures, statistically significant pair-wise comparisons based on levels of 0.05, 0.01, and 0.001 are reported by *, ** and ***, respectively. Red open symbols refer to predicted data. For non-linear saturation, in addition to the dimensionless parameter α, the right y-axis provides the THD@peak in percent.

Figure 2 shows detection thresholds for the four types of distortions as black filled symbols in the different panels. The abscissa represents the four room configurations: Anechoic, Small (mild reverberation), Small,2M_sep_ (mild reverberation plus two spatially separated maskers), and Large (moderate reverberation). Detection thresholds for spectral ripples are given as peak-to-valley ratio in dB, for non-linear saturation as the value of the dimensionless non-linearity parameter α (left y-axis), and the THD for the peak value of the reference signal (THD@peak) in percent (right y-axis). Discrimination thresholds for intensity-based distortions are reported as intensity JNDs in dB SPL, and spatial distortions are given as azimuth JNDs in degrees.

As shown in the upper panel of Figure 2, speech signals distorted by spectral ripples had significantly higher detection thresholds in the Anechoic (peak-to-valley ratio of 7.2 dB) than in the Large room (peak-to-valley ratio of 6.1 dB). Conversely, for non-linear saturation, listeners had significantly lower detection thresholds in the Anechoic than in the Large room with moderate reverberation. Intensity JNDs for the room configurations Anechoic, Small and Large ranged between 1.3 and 1.4 dB, while a JND of 2.6 dB was observed for Small,2M_sep_. Post-hoc comparisons showed no significant intensity JND differences between the room configurations Anechoic, Small, and Large. Similar azimuth JNDs of 1.9° and 2° were observed for the Anechoic and the Small room, while significant higher JNDs of about 11.7° and 8.9° were found for the Small,2M_sep_ and the Large room.

A 2-way repeated-measures ANOVA [*distortion* (spectral ripples, non-linear saturation, intensity, spatial), *room* (Anechoic, Small, Small,2M_sep_, Large)] showed a significant main effect of the factors *distortion*, *F*(2, 29.8) = 87, *p* < 0.001, and *room*, *F*(1.4, 20.5) = 39, *p* < 0.001. Moreover a significant two-way interaction between the factors *distortion* and *room*, *F*(2.4, 35.3) = 33, *p* < 0.001 was found. Statistically significant differences (post-hoc test) based on levels of 0.05, 0.01, and 0.001 are indicated in Figure 2 by *, **, and ***, respectively.

In summary, it can be concluded that the presence of maskers had a strong effect (*ES* = 0.86), while mild reverberation alone (Small room) had only a small effect (*ES* = 0.13), suggesting that results in anechoic conditions are transferable to conditions with mild reverberation.

### Supra-threshold quality ratings

Listener’s individual scores were averaged across test and retest. The test–retest Pearson-Correlation-Coefficient (PCC) of the data was 0.91 and 0.95 for *Effect of room* and *Effect of masker configuration*, respectively. For more details, test–retest PCCs for each of the 16 listeners are provided in Table 3.

**Table 3:** Individual test–retest PCC for each of the 16 listeners.

Listener	“Rooms”	“Maskers”	Overall
#1	0.92	0.99	0.95
#2	0.94	0.93	0.93
#3	0.88	0.97	0.91
#4	0.9	0.95	0.92
#5	0.99	0.99	0.99
#6	0.91	0.93	0.92
#7	0.88	0.93	0.9
#8	0.92	0.93	0.92
#9	0.89	0.95	0.9
#10	0.89	0.97	0.91
#11	0.9	0.93	0.91
#12	0.93	0.91	0.93
#13	0.92	0.97	0.93
#14	0.96	0.96	0.96
#15	0.89	0.93	0.9
#16	0.9	0.9	0.9

The first column refers to the listener, while the second and third columns refer to PCC scores based on audio quality ratings for the experiments *Effect of room* and *Effect of masker configuration*, respectively. The last column shows the overall PCC for both experiments.

#### Effect of room

**Figure 3:**
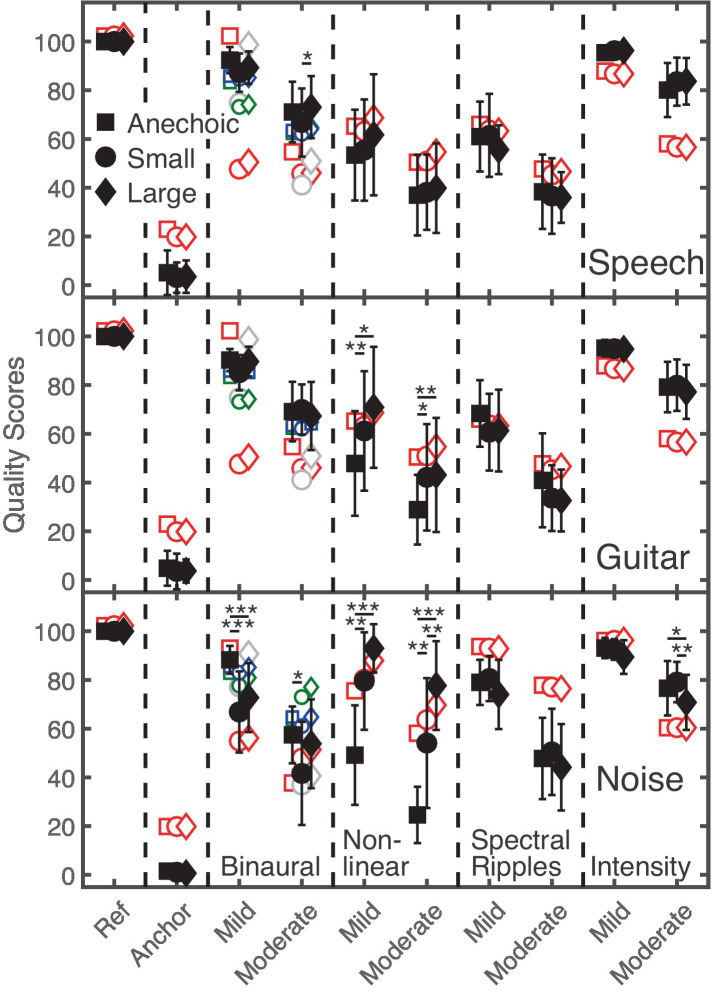
The upper, middle, and lower panels show supra-threshold audio quality ratings for speech, guitar music, and noise (black filled symbols). The ordinate represents quality scores ranging from 0 (“very strong difference”) to 100 (“no difference”). The abscissa represents the hidden reference, anchor, and type of distortion (mild and moderate amount). The Anechoic, Small, and Large room are represented by squares, circles, and diamonds, respectively. Statistically significant pairwise comparisons between rooms are indicated by the asterisks. The red and small green open symbols refer to GPSM^q^ and BAM-Q predictions, while gray and small blue symbols refer to GPSM^q^ and BAM-Q predictions for which only the direct sound and early reflections of the BRIRs were considered.

In Figure 3, the subjective quality scores (averaged across all 16 listeners; error bars indicate one inter-individual standard deviation) for speech (upper panel), guitar (middle panel) and noise (lower panel) signals impaired by spatial, non-linear, spectral, and intensity distortions are shown for the Anechoic, Small, and Large rooms, indicated by black-filled squares, circles, and diamonds, respectively. The ordinate shows the quality scores, ranging from 0 (“very strong difference”) to 100 (“no difference”). The abscissa indicates the hidden reference, anchor, and each of the four distortions having mild and moderate amounts.

A clear difference of about 21 points on the MUSHRA scale between listeners’ ratings for mildly and moderately distorted signals can be observed for each of the four distortions. The hidden reference always received the highest rating, while the anchor signal always received the lowest rating, as intended by the experimental design. For the speech target (upper panel), only slight differences in the quality scores between the three rooms were observed. A stronger effect of reverberation was observed for guitar music and noise (middle and lower panels). Here, reverberation showed a particularly strong impact on spatial and non-linear distortions. For non-linear saturation, quality ratings increased with increasing T60 and decreasing DRR. For spatial distortions, quality ratings were lower for noise presented in the Small room than in the other two rooms. Although counterintuitive, such a behavior was – to some extent - also observed for speech and guitar signals.

A 4-way, repeated-measures ANOVA [*distortions* (spatial, non-linear, spectral, intensity), *room* (Anechoic, Small, Large), *stimuli* (speech, guitar, noise), *effect strength* (mild, moderate)] showed a significant main effect of the factors *distortion*, *F*(1.5, 22) = 72, *p* < 0.001, *room*, *F*(2, 30) = 7.5, *p* < 0.01, and *strength*, *F*(1, 15) = 185, *p* < 0.001, while no significant effect was found for *stimuli*, *F*(2,30) = 2.7, *p* = 0.84.

Focusing on the effect of room in the data, only significant interactions including the factor *room* are reported: There were significant two-way interactions between the factors *room* and *stimuli*, *F*(4, 60) = 5, *p* < 0.01, and between the factors *distortions* and *room*, *F*(3.2, 48) = 39, *p* < 0.001. Moreover, three-way interactions between the factors *stimuli*, *room*, and *distortion*, *F*(4.7, 71) = 15.3, *p* < 0.001 and between the factors *room*, *stimuli*, and *effect strength*, *F*(4, 60) = 4.3, *p* < 0.01 were found.

Taken together, the room had a significant effect on quality ratings, depending on the type of distortion and the stimulus: For speech, only slight differences across the anechoic and the two echoic rooms were observed, in contrast to guitar music and noise. Thus, for the assessment of speech quality, room reverberation only appears to have a small effect. Regarding the type of distortion, quality ratings for non-linear saturation depended most strongly on the amount of reverberation.

#### Effect of masker configuration

**Figure 4:**
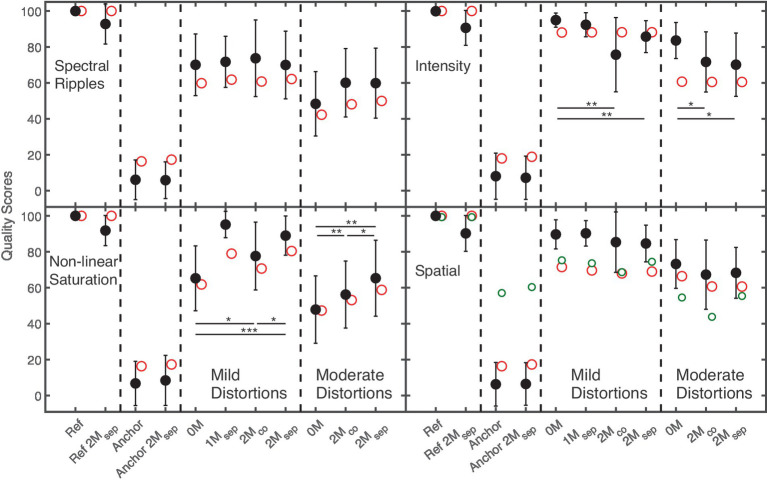
Quality ratings (black filled symbols) for the speech target with spectral ripples, intensity distortion, non-linear saturation, and spatial distortions in the Small room as a function of the masker configuration. Ref and Anchor refers to the hidden reference and the anchor signals. 0 M indicates no masker. In the configuration 1M_sep_, the ISTS masker was presented at −45° azimuth relative to the viewing direction of the listener. In configurations 2M_co_, and 2M_sep_ the ISTS and music maskers were presented at 0°, and +−45° azimuth. Statistically significant pairwise comparisons involving masker configurations 0 M, 2M_co_, 2M_sep_ are indicated by the asterisks. Red and small green open circles refer to GPSM^q^ and BAM-Q predictions.

Figure 4 shows average subjective quality scores and inter-individual standard deviations (black filled circles) for the speech target with spectral ripples (upper-left panel), non-linear saturation (lower-left panel), intensity (upper-right panel), and spatial (lower-right panel) distortions in the Small room as a function of masker configuration.

The hidden reference without maskers (Ref) always obtained the highest ratings, while the hidden reference with two spatially separated maskers (Ref 2M_sep_) received about 9 point lower scores. The anchor always received the lowest ratings, and no substantial differences between the anchors with and without maskers were observed.

The speech signal with mild spectral ripples was hardly affected by the presence of maskers, indicated by similar ratings of about 71 points. For moderate spectral ripples, a higher rating was observed for spatially co-located (2M_co_) or spatially separated (2M_sep_) maskers compared to the condition without (0 M) masker (60 vs. 48 points, respectively). However, a pairwise comparison showed no significant difference.

Lower quality scores were found for non-linear saturation without maskers than with maskers. For both mild and moderate distortions, listeners provided lower scores of about 10 points for 2M_co_ than for 2M_sep_.

For intensity distortions, higher quality scores were obtained without maskers than with maskers: Quality scores for mild intensity distortions were slightly lower (about 10 points) for co-located (2M_co_) than for spatially separated (2M_sep_) maskers, while similar quality scores (about 71 points) were obtained for moderate distortions under these two masker conditions.

The presence of maskers had only a slight effect on the perception of spatial distortions. For both mild and moderate distortions, only small differences of about 5 points between the conditions 0 M and 2M_co_, and between the conditions 0 M and 2M_sep_ were observed.

A 3-way, repeated-measures ANOVA (*distortion, effect strength, masker*) showed significant main effects of *distortion, F*(2.2, 32.3) = 14, *p* < 0.001 and *effect strength*, *F*(1, 15) = 74, *p* < 0.001, while no significant effect was found for *masker*.

Significant two-way interactions between the factors *distortion* and *effect strength*, *F*(1.9, 28.4) = 5, *p* < 0.01, and between the factors *distortion* and *masker*, *F*(2.9, 44.1) = 13.9, *p* < 0.001, were found, together with a three-way interaction between factors *distortion*, *effect strength* and *masker*, *F*(6, 90) = 3.5, *p* < 0.01. Pairwise comparisons (indicated by the asterisks in Figure 4) showed some significant effects of masker for non-linear saturation and intensity distortions. Thus, although no main effect of masker was found, quality ratings for non-linear saturation are more affected by maskers than the other distortions, as also observed for the effect of room.

### Comparison of individual results across conditions and outcome measures

To assess the relation of listener’s individual distortion detection and discrimination thresholds, a one-tailed correlation analysis was performed. The upper right side of Table 4 shows significant correlations (indicated as asterisks) between the listeners’ thresholds in the room configurations Anechoic (A), Small (S), Small,2M_sep_ (S,2M_sep_), and Large (L) for spectral ripples, non-linear saturation, and intensity distortions. Such a relationship was not observed for spatial distortions. Thus, for (monaural) spectral ripples, non-linear saturation, and intensity distortions, the listeners’ performance in anechoic, “classical psychoacoustic test” conditions might be a good indicator for their performance in echoic rooms with mild to moderate reverberation, and for acoustic environments with maskers.

**Table 4:** Statistically significant correlations for the detection and discrimination thresholds are represented as black asterisks in the upper right segment.

Distortions	Room	Spectral ripples				Non-linear				Intensity				Spatial			
		A	S	S,2M_sep_	L	A	S	S,2M_sep_	L	A	S	S,2M_sep_	L	A	S	S,2M_sep_	L
Spectral ripples	A	–	**	**	**	**	*	**	**								
	S	** *	–	**	**	**	*	**	**			*				*	
	S,2M_sep_			–	**	**		**	**			*				*	
	L	** *			–	**	*	**	**			*					
Non-linear	A	** **			* *	–		**	*			*				*	
	S	* **			* * *	** ** *	–		*	**	*	**	**				
	S,2M_sep_							–	**			*				*	
	L	* **	**		* *	** **	** **		–			**				*	
Intensity	A									–	**	**	**				**
	S	*	* *							* **	–	*	**				
	S,2M_sep_											–	**			**	**
	L		*							* ** *	** ** **		–	*			
Spatial	A	** **				*					*			–			
	S					* *	*		*						–		
	S,2M_sep_															–	**
	L	* **	** **		* **	**	*				* *			**	**		–

Significant correlations for the quality ratings for mildly distorted speech, guitar, and noise signals are shown as red, blue, and green asterisks in the lower left segment. Significance levels of 0.05, and 0.01 are reported by *, and **, respectively. Distortions and room configurations are given in the headers of the rows and columns. The abbreviations A, S, and L refer to the Anechoic, Small, and Large room, while S,2M_sep_ refers to the Small room with two spatially separated maskers.

Table 4 further indicates a significant correlation between spectral ripples and non-linear saturation. However, no relationship was found between spectral ripples and intensity distortions nor between spectral ripples and spatial distortions.

For the most complex scene in the detection experiment involving two maskers, Small,2M_sep_, significant correlations are shown in Table 4 for most of the distortions. Here, the presence of the maskers (and the corresponding masking of the target) likely dominates effects, resulting in the significant correlations.

The same correlation analysis as applied to distortion detection thresholds was also applied to the quality ratings, and is shown on the lower left side of Table 4. For clarity, only correlations for mildly distorted signals were reported in Table 4, which are comparable to those from the moderate distortions. For spectral ripples, non-linear saturation, and intensity distortion in different rooms (see Figure 3), a similar correlation pattern as observed for detection thresholds was found for speech and guitar music, but not for noise signals.

Quality ratings (see Figure 4) under conditions without maskers (0 M), with two co-located (2M_co_) and separated maskers (2M_sep_) more often revealed significant correlations between the four types of distortions and distortion strength (mild, moderate) within a certain masker configuration, than between the different masker configurations (not shown). This indicates that for a certain masker configuration (e.g., 2M_co_), listeners provided consistent individual ratings across the different types of distortions and distortion strength, but not across different masker configurations. This observation agrees with the significant correlation found between individual detection and discrimination thresholds for each of the four distortions in the condition Small,2M_sep_, and suggests that the perception of distorted signals in CAEs may depend on the individual ability to separate the distorted speech target from the maskers.

**Figure 5:**
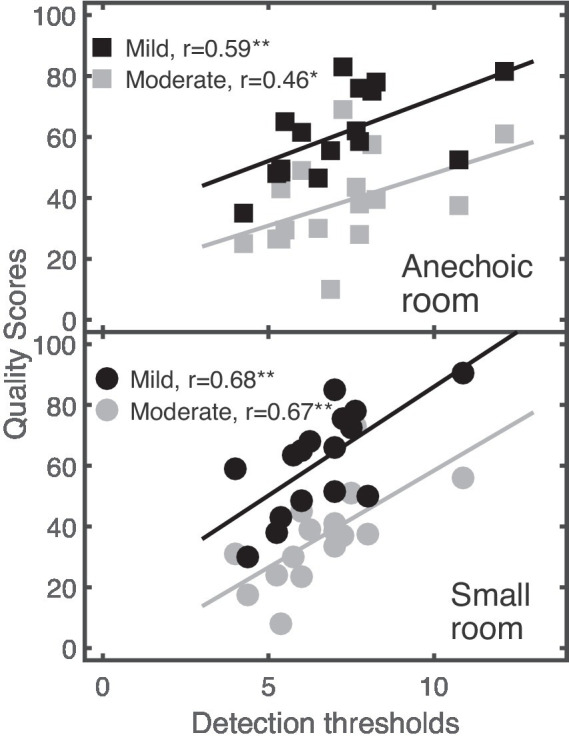
Relationship between listeners’ quality scores (ordinate) and detection thresholds (abscissa) for linear distortions in the speech target in the Anechoic and Small rooms (upper and lower panel). Mild and moderate distortions are represented by black and gray symbols, respectively. Linear regression models fitted to data are shown as solid lines. Statistically significant effects based on levels of 0.05, and 0.01 are indicated as * and **.

A one-tailed correlation analysis was used to examine a potential relationship between the listeners’ performance in the detection/discrimination thresholds and the supra-threshold quality ratings. A significant correlation was only found for spectral ripples, as indicated in Figure 5 that shows the individual quality scores as a function of the detection thresholds for the Anechoic (upper panel) and the Small room (lower panel). Quality scores increased with increasing detection thresholds, indicating that (sensitive) listeners provided lower quality scores than listeners with higher detection thresholds.

## Applicability of auditory models to CAEs

The application of (reference-based) auditory models is a common way to assess the contribution of energetic and amplitude modulation masking. Here, the GPSM (Biberger and Ewert, 2017), which has been shown to account for several psychoacoustic detection and masking experiments with less complex stimuli (e.g., pure tones and noise), was used as monaural auditory model for predicting data from the detection and discrimination experiments. The monaural audio quality model GPSM^q^ (Biberger et al., 2018), which previously successfully predicted subjective quality ratings for different types of distortions and stimuli (see also, Fleßner et al., 2019; Biberger et al., 2021), was applied to the quality ratings. Both models are based on short-term power and envelope power SNRs. Additionally, the binaural auditory model for audio quality (BAM-Q; Fleßner et al., 2017), based on the binaural psychoacoustic model front end of Dietz et al. (2011), was applied for the spatial distortion. BAM-Q predictions are based on the combination of the sub-measures ILDs, ITDs, and interaural vector strength. The same AFC-framework (Ewert, 2013) and the stimuli as used for the detection and discrimination measurements were also used for GPSM simulations. For audio quality predictions, the same sound files presented to the listeners during the audio quality rating experiments were provided to the quality models, whereas the left and right ear channels were concatenated to a one-dimensional vector when the monaural GPSM^q^ was applied. For quality predictions with maskers, an additional preprocessing step was introduced that removed signal parts with a signal-to-noise ratio (SNR) below −10 dB. The preprocessing had perfect *a-priori* knowledge about the target and the masker signals, similar to the assumption in models, to form an ideal binary mask (IBM; e.g., Wang, 2005; Brungart et al., 2006) to examine the consequences of energetic masking on speech intelligibility. A linear transformation was applied, to map the predicted quality scores onto the same scale, ranging from 0 (“very strong difference”) to 100 (“no difference”), as used for listener ratings. In Figures 2–4, predictions of GPSM and GPSM^q^ are shown as red open symbols, whereas BAM-Q predictions are shown as small green open symbols.

### Detection and discrimination thresholds

The GPSM captures only monaural cues, and therefore no predictions are shown for the spatial distortions. For spectral ripples, lower thresholds were predicted for the Small and the Large room than for the Anechoic room, similar to measured thresholds. For non-linear saturation, higher thresholds were predicted for the Large room than for the Anechoic and Small room, also in agreement with the measured data. Predicted intensity JNDs showed no systematic differences between the Anechoic, Small and Large rooms. With the exception of the predicted threshold for speech with spectral ripples in small,2M_sep_, model predictions consistently showed lower thresholds and smaller JNDs than measured data. Despite this, generally the higher sensitivity, GPSM-based predictions captured most of the room- and masker related consequences on thresholds and JNDs for those distortions. Accordingly, it is expected that the GPSM is generally applicable for the prediction of distorted speech signals in CAEs, while the higher sensitivity hints in the direction of (higher-level) cognitive effects not covered by the modeled energetic- and amplitude-modulation masking.

### Supra-threshold quality ratings

Regarding the effect of room, GPSM^q^ scores for speech, guitar and noise signals with mild distortions were always higher than with moderate distortions (see Figure 3). Monaural GPSM^q^ quality predictions for binaural distortions in the speech and guitar signals largely agree with listener’s quality ratings in the anechoic condition, but not with those in the echoic Small and Large rooms. In these monaural predictions only spectral differences were taken into account: The change of the target source position of 4° azimuth relative to the reference position (0° azimuth) resulted in large spectral differences for the echoic conditions in GPSM^q^, related to differences in the sound-reflection patterns and late reverberation for the two target source positions. The similarity of the listeners’ quality scores under those conditions suggests that only differences in the (unaltered) direct sound were considered by the listeners, ignoring the effects of reverberation. This was tested by considering only the direct sound (and early reflections) of the BRIRs in the model, which is conceptually similar to the approaches used by, e.g., Rennies et al. (2014) and Leclere et al. (2015) to simulate the effect of reverberation on (binaural) speech intelligibility by separating the early (useful) from the late (detrimental) room reflections. Gray open symbols in Figure 3 represent GPSM^q^ predictions using a 5-ms window, starting with the direct sound. This modification clearly improved prediction accuracy for the mild spatial distortions and did not degrade prediction accuracy for moderate spatial distortions. For moderate spatial distortions, it can be expected that monaural spectral differences would have had only a minor effect on listeners’ quality ratings. Binaural predictions were also improved when using the same 5-ms window (small blue symbols), less, however, than for the monaural predictions.

For non-linear distortion, there are more pronounced differences in the listeners’ quality ratings between room conditions than observed in predicted scores, particularly for guitar and noise signals. GPSM^q^ mainly predicts higher scores for speech in the Large room than in the other two rooms. Measured and predicted quality scores of speech, guitar and noise signals with spectral ripples showed no substantial effect of room reverberation. Similarly, no room dependence was observed in the measured and predicted quality scores for intensity distortions in speech and guitar signals. A room effect was only observed for noise signals with moderate intensity distortions, where listeners provided significantly higher ratings for the Anechoic and Small rooms, than for the Large room. Such differences were not observed by GPSM^q^ prediction. Given that the intensity distortions cause loudness differences between the reference and the distorted signal, loudness models (e.g., Chalupper and Fastl, 2002; Pieper et al., 2018) may account for the observed differences. However, loudness predictions (not shown) of the dynamic loudness model (DLM; Chalupper and Fastl, 2002) provided a similar loudness ratio between reference and test signal of about 1.3 in the three rooms used, suggesting similar perceived loudness differences. Thus, neither the GPSM^q^ nor the DLM predicted the observed effect of room for moderate intensity distortions with noise. Despite such deviations, GPSM^q^ achieved an overall good prediction performance for audio quality of distorted signals in rooms with different reverberation times, indicated by a Pearson correlation coefficient of 0.8 and a Spearman rank correlation coefficient of 0.75.

For the different masker configurations (speech target), predicted quality scores of GPSM^q^ are shown in Figure 4. The preprocessing only kept unmasked and thus reliable time segments of the target. The predicted quality scores for anchor signals and spectral ripples, non-linear saturation, and spatial distortion agree well with the data. The preprocessing assumes that masked segments of the distorted target do not affect the listeners’ quality ratings of the entire distorted signal. Given the accurate predictions, this assumption appears to be valid for anchor signals and spectral ripples, non-linear saturation, and spatial distortion, suggesting a certain degree of invariance of the perceptual quality attributes of the target auditory object in the presence of maskers. For intensity distortions, lower quality scores were obtained with maskers than without maskers by the listeners, whereas similar scores were predicted with and without maskers. For intensity distortion, listeners’ quality ratings are likely based on a comparison between the target loudness of the reference and the test signal. Hypothetically, the reduced number of spectro-temporal segments (or observations) of the target available to the auditory system in the presence of the masker decreases the perceived target loudness. A comparable effect of maskers on the target loudness was also observed by Fichna et al. (2021), where the loudness of the target speaker decreased with an increasing number of maskers. Consequently, target loudness (as the presumably underlying quality attribute for the intensity distortions) is invariant in the presence of other interfering auditory objects and masking effects have to be taken into account (see upper right panel of Figure 4). Overall, GPSM^q^ predictions agreed well with subjective quality ratings for distorted signals in the presence of maskers, indicated by a Pearson correlation coefficient of 0.87 and a Spearman rank correlation of 0.89. Binaural BAM-Q predictions for the spatially distortions show a similar pattern as observed in the measurements. Surprisingly, BAM-Q predicted higher quality scores for the anchor signal (target position at 40°) than for the speech target with moderate spatial distortions (target position at 30°). While BAM-Q observed larger ITD differences for the 40° target position than for 30°, lower ILD differences were observed, with no substantial differences for IVS. The final quality measure provided by BAM-Q was obtained by combining ILD, ITD, and IVS differences, with ILDs receiving the strongest weighting (see Section “Applicability of auditory models to CAEs” in Fleßner et al., 2017), thus explaining the surprisingly high quality ratings for the anchor signal.

## Discussion

### Detection and discrimination thresholds

No statistically significant differences between the Anechoic and the Small room occurred for the detection thresholds of the four distortions, suggesting that anechoic thresholds are also representative for rooms with mild reverberation (T60: 0.35 s), as typically encountered in home environments. Conversely, with the exception of intensity distortion, significant threshold differences were found between the Large room with moderate reverberation (T60: 1.5 s) and the other two rooms. The absence of any effect of intensity distortion can be expected, given that neither spectral, nor amplitude modulation, nor spatial changes were introduced. All room acoustic features, such as the pattern of early reflections and the DRR, were invariant to the level changes introduced in the intensity distortion.

For spectral ripples, one reason for lower thresholds in the Large room might be an improved audibility of spectral ripples in certain frequency regions because of the room’s modal structure. Similarly, Toole and Olive (1988) observed a better detectability of signal resonances in echoic than in anechoic rooms, which was presumably a result of an improved audibility of such resonances. According to the representation of power-based SNRs in the auditory model GPSM, the most dominant spectral differences between the Anechoic and the Large room occurred below 800 Hz.

Non-linear saturation resulted in additional frequency components at higher signal levels, which likely provided spectral or amplitude modulation cues to the listeners. A comparison of the power- and envelope-power SNR representation (across auditory and modulation filters) showed increased energy between 2 kHz and 3.15 kHz for non-linear saturation under anechoic conditions. Particularly large differences were observed in high modulation filters (above 64 Hz) at signal onsets. Such differences were substantially reduced with moderate reverberation in the Large room.

Substantially increased position JNDs for the target in the Large room suggest a degradation of binaural cues in the signal onsets. For sound localization, e.g., Wallach et al. (1949), Blauert (1971) have shown that the direction of the sound that arrives at the ears first dominates perception compared to later-arriving reflections from other directions. Accordingly, signal onsets are important for sound localization in real rooms (e.g., Stecker and Moore, 2018), as the onsets may be less impaired by overlap masking. To interpret the current results, a binaural auditory model (Dietz et al., 2011) was also applied here (not shown). Only the direct sound and early reflections up to 50 ms after the direct sound were analyzed, reflecting a simplistic simulation of the precedence effect motivated by, e.g., Haas (1972) and Lochner and Burger (1964). Consistently pointing ILDs (> 1,500 Hz) and ITDs (< 1,500 Hz) were found for the Anechoic and Small rooms, but more strongly fluctuating ITDs were found for the Large room. Only slight differences in ILDs (> 1,500 Hz) were observed between the Small and the Large rooms, suggesting that ITDs served as a main cue under the current room conditions.

Maskers (here the interfering ISTS speech signal and pop music) caused a substantial increase of detection and discrimination thresholds for all four distortions. As supported by the model simulations, this is a direct consequence of the reduced amount of distorted spectro-temporal speech segments available to the listeners, hampering the detection of distortion effects in the target. Thus, particularly for CAEs with mild reverberation, the effect of masking caused by interfering sounds is most relevant.

The correlation analysis for listener’s individual thresholds (see Section “Comparison of individual results across conditions and outcome measures”) indicated that well-performing listeners, who obtained low detection thresholds for linear, non-linear saturation and intensity distortions in the Anechoic room, mostly remained good performers in the echoic rooms with and without maskers. Conversely, this was not observed for spatially distorted speech. Overall, findings of the correlation analysis suggest that for spectral ripples, non-linear saturation, and intensity distortion, the individual listener’s performance under anechoic conditions might be a good indicator for their performance in CAEs with mild- to moderate reverberation and maskers, but not for spatial distortion.

### Supra-threshold quality ratings – Effect of room

Overall, the supra-threshold perception of distortions was affected by reverberation, as supported by the significant main effect of *room* on quality ratings. However, the effect depended on the stimulus and the type of distortion, as indicated by the significant interactions reported in Section “Effect of room”:

Quality ratings for spectral ripples were hardly affected by reverberation. Although no significant effects were found, the trend that for all three stimuli, spectral ripples were rated higher in the Anechoic room than in the Small and Large rooms, agrees with the effects found in the detection experiment.

For signals distorted by non-linear saturation, higher quality ratings were obtained in reverberation than in the Anechoic room. Here, as observed for detection thresholds, reverberation is expected to mask distorted parts of the signals. As shown in Figure 3 and indicated by the interaction between factors *stimuli* and *room*, non-linear saturation in guitar music and noise was more effectively masked by reverberation than in the speech signal. This is presumably based on differences in the signal properties of the fluctuating speech, and guitar signals and the stationary noise signal: Non-linear saturation mainly affects signal peaks in fluctuating targets, which provide high SNRs in reverberation, while harmonic distortions in noise mainly result in perceivable spectral coloration changes.

For intensity distortion, room reverberation had no effect on the listeners’ quality ratings, except for moderate intensity distortion in noise. Here, the lower quality ratings in the Large room compared to the Anechoic and Small rooms, imply larger perceived differences. The dominating supra-threshold cue associated with intensity is loudness. Accordingly, a loudness model (Chalupper and Fastl, 2002) was applied in Section “Applicability of auditory models to CAEs,” but did not explain the lower quality ratings in the Large room for that specific condition (see lower panel in Figure 3), but agreed with the other quality ratings for intensity distortions. Overall, for intensity distortions it can be summarized that reverberation had no, or only a minor, effect on quality ratings as already observed for intensity JNDs (see Figure 2).

For spatially distorted noise signals, lower quality ratings were obtained in the Small room than in the Anechoic and Large rooms. This appears counterintuitive, given that a smaller effect of reverberation would be assumed for the Small than for the Large room. Here, listeners may have rated spectral differences instead of spatial differences: A comparison of the (third-octave-smoothed) frequency spectra of the noise target at 0° and 4° in the Small room shows level differences between frequencies of 850 Hz to 1,440 Hz of up to 3 dB, while only slight level differences were observed in the Large room. Therefore, spectral as well as binaural cues appear relevant for the perception of spatial distortions, depending on the specific echoic environment.

A central question of this study was if listeners who showed lower detection thresholds than other listeners were also more sensitive in the quality ratings than the others. Such relationship would allow making individual adjustments in, e.g., hearing devices, purely based on distortion detection thresholds. The correlation analysis revealed a significant correlation between detection thresholds and quality ratings for speech signals with spectral ripples. Therefore, information about the listener’s threshold for spectral ripples might be sufficient for an individualized adjustment of hearing devices concerning spectral ripples when focusing on speech quality.

### Supra-threshold quality ratings – Effect of masker configuration

Based on the models applied in this study and the concept of energetic masking, it is expected that listeners base their quality judgements on reliable (unmasked) spectro-temporal segments of the distorted target in the presence of fluctuating maskers. For equally distributed distortions over time, it thus appears plausible to expect only slight differences between quality ratings with and without maskers, given that the effect of distortion is observable in the unmasked spectro-temporal segments. For non-equally distributed distortions, differences can be expected when, e.g., more-strongly distorted segments are masked, while mildly distorted segments are not masked. Such a behavior was observed for (moderate) spectral ripples and non-linear saturation where listeners rated quality higher for the masked than for the unmasked distorted target (speech) as shown in Figure 4. Here, saturation distortions considered in this study were unequally distributed over time, as they only occurred at higher signal levels. Although the spectral ripples applied in this study are in principle equally distributed over time, the spectral composition of the target changed over time, and thus provided spectro-temporal segments where the distortions were easier to detect than in other segments. This interpretation agrees well with the quality predictions shown in Figure 4, where audio quality was estimated using only reliable and unmasked segments of the distorted target (with an SNR > = −10 dB).

For intensity or spatial distortions, the presence of maskers lowered the perceived quality. Intensity distortions were introduced by decreasing the overall level of the target. Therefore, in the quality ratings for intensity distortions, listeners likely rated loudness in comparison to the reference. Accordingly, the observed lower scores in the presence of maskers might reflect a lower perceived loudness of the target, as parts were masked and not accessible to the listeners. A similar observation was made in Fichna et al. (2021), where the loudness of a target speaker was decreased as the number of the maskers was increased. A masker-induced loudness reduction was also observed in the data of a “classical” loudness experiment presented in Figures 8–10 in Fastl and Zwicker (2006) where the loudness of a 1-kHz tone was reduced as a stationary pink-noise masker was added to the tone.

As for intensity distortions, a slight tendency for lower quality ratings in the presence of maskers was also observed for spatial distortions. Surprisingly, no difference between quality ratings was observed for co-located and spatially separated maskers. Here, it might be expected that the target at 4° azimuth (mild spatial distortion) was more efficiently masked by co-located maskers (at 0°) than by separated maskers (at ±45°), while the moderate spatial distortion at 30° azimuth was more efficiently masked by the separated maskers than by co-located maskers. However, the diversity of the ISTS and pop-music maskers and the speech target may have facilitated segregation and direction estimation of these perceptually very different sound sources.

Another interesting effect was observed for the quality ratings assigned to the reference with and without maskers. On average, listeners rated the reference with maskers (2M_sep_) 9 points lower than without maskers. Here, the maskers likely introduced an uncertainty about the reference and affected the overall rating. Only one listener ignored the maskers and provided a rating of 100 points. This uncertainty effect is an important finding for reference-based audio quality predictions, as quality differences between the reference with and without maskers cannot simply be predicted by only taking unmasked spectro-temporal segments of the reference signal into account (which would not predict any quality difference). Accordingly, for audio quality models, an uncertainty has to be considered, which may depend on the spatial position of the masker, the number and the type of maskers.

### Implications for auditory models

Detection and discrimination thresholds were more accurately predicted than quality ratings, showing that basic sensory cues are reasonably well represented in the model’s auditory preprocessing. As shown in Figure 2, GPSM consistently predicted lower thresholds and JNDs than observed in the data. Such higher sensitivity of the model compared to the listeners could be reduced by introducing additional internal noise as suggested in earlier studies (Dau et al., 1997; Wallaert et al., 2017; Ewert et al., 2020) to represent further cognitive effects, which might be related to segregation of the target from the scene.

While GPSM^q^ captured most of the effects of reverberation on quality ratings, it strongly overestimated the spectral differences related to differences in the sound reflection patterns between target positions of 0° and 4°. GPSM^q^ predictions can be improved when only the direct sound and very early reflections of up to 5 ms are analyzed, both considered as “useful,” whereas late room reflections are considered as masker (“detrimental”). The same 5-ms temporal window also improved binaural quality predictions of BAM-Q for spatially distorted speech signals in echoic rooms. The underlying cognitive effects of separating and segregating direct sound and (typically correlated) early sound reflections, from typically uncorrelated late reverberation, representing a background “masker,” have to be considered for future modeling.

For quality predictions in the presence of maskers, a preprocessing was applied to the waveform of the signals, removing “unreliable” temporal segments with an SNR below −10 dB. In contrast to the data, without such a preprocessing, GPSM^q^ would predict higher quality scores for conditions with maskers, because the model would observe reduced differences between the test and reference signal for temporal segments dominated by the masker. As shown in Figure 4, quality predictions of GPSM^q^ with preprocessing for spectral ripples, non-linear saturation, and spatial distortion agreed well with data; they did not, however, capture the effect of maskers for intensity distortions. Here, instrumental measures would have to predict an apparent lower overall target loudness for acoustic environments with maskers than without maskers.

## Summary and conclusion

Detection thresholds and supra-threshold audio quality ratings of spectral ripples, non-linear saturation, intensity, and spatial distortions of a target in complex acoustic environments was investigated. The complexity of the environments was changed by varying the number of maskers and the amount of reverberation. Speech served as the main target in all conditions, while the effect of reverberation was additionally examined for a guitar and pink-noise target. The following conclusions can be drawn:

- Detection thresholds for distorted speech targets in anechoic and mild reverberation showed no significant differences, suggesting that findings in anechoic conditions are transferable to conditions with mild reverberation. Conversely, a significant effect of moderate reverberation on detection thresholds for spectral ripples, non-linear saturation, and spatial distortion was found, indicating the relevance of additional measurements with moderate reverberation when assessing performance in CAEs.
- Reverberation showed only a small effect on quality ratings for distorted speech, but had a stronger effect on guitar and noise signals. This effect is presumably based on differences in the signal properties of the fluctuating speech, guitar music and the stationary noise, that changes the sound character of the distortions.
- Increased detection thresholds for distorted speech in the presence of two maskers were measured compared to the situations without masker. The effect of maskers on quality depended on the type of distortions. In connection with the model analysis, it appears that quality ratings were based on unmasked temporal speech segments.
- A significant correlation between listeners’ individual detection thresholds and their quality ratings for spectral ripples in speech targets was found. Sensitive listeners with low detection thresholds also provided lower quality scores than listeners with higher detection thresholds.
- The GPSM (Biberger and Ewert, 2017) and the GPSM^q^ (Biberger et al., 2018), captured the main effects of CAEs on detection thresholds and quality ratings in different room- and masker configurations, indicated by Pearson linear-correlation coefficient values of 0.8 and 0.87, respectively. For accurate quality predictions in the presence of maskers, a preprocessing that only provided “reliable” speech segments to GPSM^q^ was required.

## Data availability statement

The raw data supporting the conclusions of this article will be made available (www.faame4u.com) by the authors without undue reservation.

## Ethics statement

The studies involving human participants were reviewed and approved by the Ethics Committee of the University of Oldenburg. The participants provided their written informed consent to participate in this study.

## Author contributions

TB and SE co-conceived the ideas. SE supervised the project. TB carried out the experiments and model simulations. All authors contributed to the article and approved the submitted version.

## Funding

This work was supported by the Deutsche Forschungsgemeinschaft (DFG – 352015383 – SFB1330 A2 and DFG –390895286 – EXC 2177/1).

## Conflict of interest

The authors declare that the research was conducted in the absence of any commercial or financial relationships that could be construed as a potential conflict of interest.

## Publisher’s note

All claims expressed in this article are solely those of the authors and do not necessarily represent those of their affiliated organizations, or those of the publisher, the editors and the reviewers. Any product that may be evaluated in this article, or claim that may be made by its manufacturer, is not guaranteed or endorsed by the publisher.
